# *Streptococcus pneumoniae* and influenza vaccination rates in oncological patients — data from Germany

**DOI:** 10.1007/s00520-024-09023-y

**Published:** 2024-11-21

**Authors:** Emma Niederstein, Journey Underwood, Maike de Wit, Mark Reinwald, Sandra Schwarzlose-Schwarck, Werner Dammermann, P. Markus Deckert, Til Ramón Kiderlen

**Affiliations:** 1 Faculty of Medicine and Psychology, Brandenburg Medical School Theodor Fontane, 14770 Brandenburg an der Havel, Germany; 2 Department of Hematology, Oncology and Palliative Care, Vivantes Hospital Neukoelln, 12351 Berlin, Germany; 3 Department of Oncology, Vivantes Auguste-Viktoria-Hospital, 12157, Berlin, Germany; 4 Department of Hematology and Oncology, University Hospital Brandenburg an der Havel, 14770 Brandenburg an der Havel, Germany; 5 Faculty of Health Sciences Brandenburg, Brandenburg Medical School Theodor Fontane, 14770 Brandenburg an der Havel, Germany

**Keywords:** *Streptococcus pneumoniae*, Influenza, Vaccination rates, Cancer patients, Oncology, Hematology, Immunosuppression

## Abstract

**Objectives:**

Due to disease- or therapy-associated immunosuppression, oncological patients suffer from significantly higher morbidity and mortality due to infections transmitted by respiratory pathogens such as *Streptococcus pneumoniae* and influenza virus. Although the German Standing Committee on Vaccination (STIKO) provides specific recommendations for vaccination against these pathogens, there is no data on vaccination rates in this high-risk population.

**Methods:**

Data from the interventional *EVO study* were analyzed to provide information on vaccination rates against *Streptococcus pneumoniae* and influenza virus in oncological patients. Numbers presented in this publication summarize baseline and follow-up data of the control group; thus, data were not influenced by the intervention.

**Results:**

Data of 370 patients were analyzed; 20.5% of patients were treated for hematological malignancies and 79.5% for solid cancer. 28.1% of patients had received vaccination against influenza and 32.2% against *Streptococcus pneumoniae*; for the latter only 7.3% according recommendations. While vaccination rates where even lower for patients with thoracic carcinoma (influenza 26.7% and *Streptococcus pneumoniae* 6.0% according to STIKO recommendations), rates in patients with multiple myeloma were remarkably higher (39.0% and 14.6%).

**Conclusions:**

Despite strong recommendations to vaccinate and the clear clinical need to prevent infections in the vulnerable group of oncological patients, only the minority was vaccinated against *Streptococcus pneumoniae* or influenza, underlining the urgent need for better vaccination strategies in this high-risk population.

## Introduction

Oncological, including hematological, patients have a significantly higher morbidity and mortality related to infections than the general population based on immunosuppression associated with the malignancy or induced by therapy [1]. Vaccinating this group of patients therefore has the potential to substantially reduce the burden of disease for many serious infections in this high-risk population [2]. Although in Germany general practitioners (GP) are responsible for vaccinating patients, they are often hesitant to vaccinate oncological patients because of uncertainness regarding implications for underlying disease and treatment [3]. Indeed, in a survey of French GPs, only 37% stated they vaccinated all of their chemotherapy patients as per recommendations and the majority of those GPs who did not stick to these recommendations voiced concerns about not being properly trained and the lack of available information on vaccination in this population [4]. A recent Italian survey showed similar limitations regarding knowledge, awareness, and attitude in oncologists [5]. On the other hand, vaccination schedules are typically not perceived as one of the tasks of oncologists; therefore, they mostly do not check and provide vaccinations for their patients [6]. This might contribute to generally lower vaccination rates in oncological patients than in the general population [7].

For *Streptococcus pneumoniae* and influenza virus, this gap potentially results in serious consequences. About 20% of severe pulmonary infections in oncological patients are related to *Streptococcus pneumoniae* [8–10], and yet about 70% of invasive pneumococcal disease (IPD) cases are caused by serotypes possibly preventable by vaccination [8, 9, 11]. Also hospitalization rates related to influenza infections are four times higher in patients with hematological and oncological diseases than in the general population while mortality rates are even ten times higher [12, 13].

At the time of our study, the German Standing Committee on Vaccination (STIKO) recommended sequential vaccination against *Streptococcus pneumoniae* with 13-valent pneumococcal polysaccharide vaccine (PCV13) followed by the 23-valent pneumococcal polysaccharide vaccine (PPV23) for oncological patients [11]. Since September 2023, the 20-valent conjugate vaccine (PCV20) as single shot replaced the former recommendation STIKO [14]. Furthermore, seasonal influenza vaccination with an inactivated quadrivalent vaccine is recommended, for the elderly as high-dose vaccination [15–17].

Patients receiving immunosuppressive treatment should ideally be vaccinated before the beginning of chemotherapy [18], if possible. Nonetheless, if vaccination is required during chemotherapy, recommendations suggest administration at the beginning of a chemotherapy cycle as there might be a better response than during the course, even though little data is available regarding the best timing [7, 19, 20]. As patients receiving T- and B-cell-depleting therapies (e.g., rituximab) do not respond to vaccinations, it is not recommended to vaccinate these patients during and at least 6 months after chemotherapy [15, 16]. Revaccinations after chemotherapy are recommended as there may be a loss of immunity due to treatment [21].

Taken the unsatisfactory low vaccination rates in the general population, there are very little data for oncological patients in Germany [7, 19, 20]. Therefore, providing numbers is of utmost importance to assess the risk and plan interventions to increase vaccination rates.

## Methods

### Study design

Data from the Easy Vaccination in Oncology (EVO) study, an interventional study assessing the impact of an intervention intending to increase vaccination rates among oncological patients for a specific set of recommended vaccines, were analyzed for vaccination rates of influenza and *Streptococcus pneumoniae* [22]. The study was conducted from January 02, 2020, to January 31, 2021, at four outpatient clinics in different districts of Berlin. Eligible participants were oncological patients, including those with hematological malignancies, undergoing antineoplastic treatment [6].

### Patient recruitment

Patients aged 18 years and older with a confirmed oncological diagnosis undergoing oncological therapy at time of the initial interview (before the intervention) or follow-up (3 months after the intervention) were included if they consented to participate in the study. Patients with known intolerances to scheduled vaccinations or who were undergoing B- and T-cell-depleting treatment were not included in the study due to the risk of an allergic reaction and the lack of possibility to be actively immunized.

### Data collection and processing

To ensure data accuracy was not affected by the intervention, our analysis included only patients from the baseline survey and from the follow-up control group, not the follow-up intervention group. Verification of vaccination status was achieved through an examination of vaccination cards. If a vaccination card was non-existent, we classified the individual as “not vaccinated” in accordance with international guidelines.

In the case of vaccinations against *Streptococcus pneumoni*ae, adherence to 2020 STIKO recommendations was determined when patients had either received sequential vaccinations with PCV13 and PPV23 or had received a PCV13 vaccination within the last 6 months, with the assumption of subsequent completion with PPV23. In this study, patients who had received influenza vaccination with STIKO-recommended vaccine during the 2018/2019 or 2019/2020 season were considered vaccinated.

Vaccination rates were compared between different diseases, age, gender, and treatment of patients, as well as between each other. To determine the vaccination rates in relation to the age of the patients, they were divided into age groups (age ≤ 59 years; age ≥ 60 years). The division of the age groups was based on the general STIKO recommendation for persons aged 60 years and older.

Main diagnoses were documented and grouped to analyze the potential impact on vaccination. Grouping was done as follows: acute leukemia (acute lymphoblastic leukemia, acute myeloid leukemia), chronic leukemia (chronic lymphocytic leukemia), lymphoma (non-Hodgkin lymphoma, Hodgkin lymphoma), multiple myeloma, other hematologic disorders (myelodysplastic syndrome, aplastic anemia, antibody deficiency), endocrine carcinoma, urological carcinoma (prostate carcinoma, urothelial carcinoma, penile carcinoma, testicular carcinoma, renal cell carcinoma), gynecological carcinoma (tubal carcinoma, endometrial carcinoma, ovarian carcinoma, cervical carcinoma), gastrointestinal carcinoma (rectal carcinoma, sigmoid carcinoma, colon carcinoma, esophageal carcinoma, gastric carcinoma, anal carcinoma, bile duct carcinoma, pancreatic carcinoma, hepatocellular carcinoma), skin carcinoma (malignant melanoma, merkel cell carcinoma), thoracic carcinoma (non-small cell lung carcinoma, small cell lung carcinoma, breast cancer, pleural mesothelioma), laryngeal carcinoma (tonsillar carcinoma), and other solid tumors (thymic carcinoma, glioblastoma, angiosarcoma).

Secondary diagnoses (SD) were documented and grouped to analyze additional reasons for vaccination. Grouping was done as follows: cardiac SD (hypertension, arrhythmia, post myocardial infarction, coronary artery disease, heart failure, cardiomyopathy), pulmonary SD (chronic obstructive pulmonary disease/COPD, bronchial asthma, interstitial lung disease), renal SD (renal failure), vascular SD (thrombosis, embolism, thrombocytopathy, coagulation disorders), gastrointestinal SD (colitis, chronic, pancreatitis, chronic cholecystitis), hepatic SD (hepatitis), endocrine SD (diabetes mellitus, hyper- or hypothyroidism), and autoimmune SD (rheumatic diseases, arthritis).

### Statistical analysis

To analyze baseline variables, standard descriptive statistics were used. Vaccination rates were compared between different diseases, age, and gender of patients, as well as between each other. For this purpose, means, minima and maxima, percentages, and exact counts were determined. The Kolmogorov–Smirnov and the Shapiro–Wilk test were performed to test the normal distribution of the variables. Data found to be not normally distributed were examined using Pearson’s chi-squared tests, Cramér’s V, Phi, and a likelihood quotient test for categorical variables. For continuous variables, binary logistic regression analyses were used. A two-sided *p*-value of *p* ≤ 0.05 served as an indicator of significance for all performed tests. Visualization was done through various tables. The IBM SPSS Statistics 29 software package was used for all statistical analysis.

### Registration

The study was registered at the German Register for Clinical Studies (DRKS) under the number DRKS00020118.

## Results

### Main characteristics of study population

A total of 370 patients were included in the analysis. Gender was equally distributed (50.8% female vs. 49.2% male). Mean age was 68 years (28–92), with 94.1% being 50 years and older. 20.5% of patients were treated for hematological malignancy and 79.5% for solid cancer. 27.3% could not provide a vaccination card and thus were documented as “not vaccinated,” following WHO recommendations.

### Vaccination rates depending on vaccine

The majority of patients were not vaccinated with any vaccine against *Streptococcus pneumoniae* (see Table 1). 32.2% of patients had received some vaccination against *Streptococcus pneumoniae*, but only 7.3% of patients were found to be sequentially vaccinated according to STIKO recommendations (including six patients having received PCV13 less than 6 months ago). Rate for vaccination with PPV23 alone was with 20.0% substantially higher, but found only in the group of patients of age 60 years and older.

**Table 1:** Vaccination rates against *Streptococcus pneumoniae* in oncological patients with different vaccines in frequency (*n*/*N*) and percentage (%)

	Vaccination status *n*/*N* (%)
Not vaccinated	251/370 (67.8%)
Only PPV23	74/370 (20.0%)
Only PCV13	23/370 (6.2%)
PCV13 + PPV23 (sequential)	22/370 (5.9%)
Vaccinated according to STIKO recommendations	**27/370 (7.3%)**

28.1% of the study population were vaccinated against influenza.

### Vaccination rates depending on gender and age

Vaccination rates against *Streptococcus pneumoniae* showed a propensity for female patients compared with male patients (9.6% vs. 4.9%; *p* = 0.087). Influenza vaccination rates presented similar results (32.4% vaccinated female patients vs. 23.6% vaccinated male patients; *p* = 0.061).

Eighty-eight patients (23.8%) were under 60, while 288 patients (76.2%) were 60 or older. Patients under 60 years of age received slightly more often sequential vaccinated according to STIKO recommendations than patients over 60 years of age (9.1% vs. 6.7%; *p* = 0.459). Vaccination rates for PPV23 alone were with 20% remarkably higher, but found only in the group of patients aged 60 years and older. No dependence of the vaccination against *Streptococcus pneumoniae* on the patients’ age could be proven (*p* = 0.564). In contrast, a significant dependence on the patients’ age was demonstrated for influenza vaccination (*p* = 0.025). Patients over 60 years of age were vaccinated against influenza more frequently than patients under 60 years of age (30.5% vs. 20.5%; *p* = 0.051).

### Vaccination rates depending on patients’ main diagnosis

Vaccination rates against *Streptococcus pneumoniae* in patients with hematological and solid cancer did not differ substantially (7.9% vs. 7.1%; *p* = 0.822) while the rates against influenza did, even though without statistical significance (35.8% vs. 26.0%; *p* = 0.137). It was particularly noticeable that in the group of hematological patients, only those with multiple myeloma had been vaccinated sequentially against *Streptococcus pneumoniae* according to STIKO recommendations (*p* = 0.055) and had a higher vaccination rate against influenza (39.5%; *p* = 0.119). For the group of solid cancer, it is noteworthy that only 6.0% of patients with thoracic carcinoma were vaccinated against *Streptococcus pneumoniae* (*p* = 0.528) and 26.7% against influenza (*p* = 0.580) as recommended. For both pathogens, these rates were lower than the corresponding overall vaccination rates (7.3% for *Streptococcus pneumoniae* (*p* = 0.288) and 28.1% for influenza (*p* = 0.364)).

The analysis of gender-specific differences in vaccination rates within the subgroup of patients with thoracic carcinoma showed the following: 8.8% of women were vaccinated against *Streptococcus pneumoniae*, while only 3.4% of men received a vaccination (*p* = 0.224).

A significantly higher proportion of women in this patient group have also been vaccinated against influenza (36.8%), while only 16.9% of men have been vaccinated (*p* = 0.016).

Vaccination rates according cancer diagnosis are found in Table 2.

**Table 2:** Vaccination rates against *Streptococcus pneumoniae* according STIKO recommendation and influenza depending on underlying cancer disease in frequency (*N*) and percentage (%)

	Hematological cancer (*N* = 76) *n* (20.5%)					Solid cancer (*N* = 294) *n* (79.5%)								Total (*N* = 370) *n* (100.0%)
	Acute leukemia (*N* = 3)	Chronic leukemia (*N* = 1)	Lymphoma (*N* = 25)	Multiple myeloma (*N* = 41)	Other (*N* = 6)	Endocrine carcinoma (*N* = 1)	Urologic carcinoma (*N* = 26)	Gynecologic carcinoma (*N* = 41)	Gastrointestinal carcinoma (*N* = 80)	Other (*N* = 6)	Skin carcinoma (*N* = 11)	Thoracic carcinoma (*N* = 116)	Throat carcinoma (*N* = 13)	
*Streptococcus pneumoniae*	0 (0.0%) (*p* = .626)	0 (0.0%) (*p* = .779)	0 (0.0%) (*p* = .146)	6 (14.6%) (*p* = .055)	0 (0.0%) (*p* = .488)	0 (0.0%) (*p* = .779)	5 (19.2%) (*p* = .015)	3 (7.3%) (*p* = .996)	6 (7.5%) (*p* = .937)	0 (0.0%) (*p* = .488)	0 (0.0%) (*p* = .345)	7 (6.0%) (*p* = .528)	0 (0.0%) (*p* = .303)	
	6 (7.9%) (*p* = .822)					21 (7.1%) (*p* = .822)								27 (7.3%) (*p* = .288)
Influenza	0 (0.0%) (*p* = .270)	0 (0.0%) (*p* = .526)	7 (28.0%) (*p* = .941)	16 (39.0%) (*p* = .119)	4 (66.7%) (*p* = .038)	0 (0.0%) (*p* = .526)	10 (38.5%) (*p* = .251)	8 (19.5%) (*p* = .314)	18 (22.3%) (*p* = .274)	3 (50.0%) (*p* = .244)	3 (27.3%) (*p* = .918)	31 (26.7%) (*p* = .580)	4 (30.8%) (*p* = .863)	
	27 (35.6%) (*p* = .137)					77 (26.2%) (*p* = .137)								104 (28.1%) (*p* = .364)

### Vaccination rates depending on patients’ secondary diagnosis

When analyzing secondary diagnosis (SD) of patients as a potential additional reason for vaccination against *Streptococcus pneumoniae* and influenza, it is noticeable that numerically more patients without prior secondary pulmonary disease were vaccinated against both pathogens than patients with prior secondary pulmonary disease (8.0% vs. 4.9% and 29.8% vs. 24.7%; *p* = 0.356 for *Streptococcus pneumoniae* and *p* = 0.373 for influenza) (Table 3). The same applies to patients with and without chronic infections (7.5% vs. 0.0% and 28.7% and 25.0%; *p* = 0.422 for *Streptococcus pneumoniae* and *p* = 0.817 for influenza). The group of patients with cardiac or endocrinological diseases showed increased vaccination rates against *Streptococcus pneumoniae* as well as influenza compared to the group of patients not suffering from these diseases (for cardiac diseases, 7.9% vs 6.5% and 31.5% vs. 24.7% *p* = 0.616 for *Streptococcus pneumoniae* and *p* = 0.153 for influenza; for endocrinological diseases, 9.0% vs. 6.7% and 33.0% vs. 26.8% *p* = 0.449 for *Streptococcus pneumoniae* and *p* = 0.238 for influenza).

**Table 3:** Vaccination rates against *Streptococcus pneumoniae* and influenza in oncological patients according to secondary diagnoses (SD) in frequency (*n*/*N*) and percentage (%)

Secondary diagnosis	SD not documented (*N*)		SD documented (*N*)	
	*Streptococcus pneumoniae* (*n*)	Influenza (*n*)	*Streptococcus pneumoniae* (*n*)	Influenza (*n*)
Cardiac	10/154 (6.5%) (*p* = .616)	38/154 (24.7%) (*p* = .153)	17/216 (7.9%) (*p* = .616)	68/216 (31.5%) (*p* = .153)
Pulmonary	23/289 (8.0%) (*p* = .356)	86/289 (29.8%) (*p* = .373)	4/81 (4.9%) (*p* = .356)	20/81 (24.7%) (*p* = .373)
Nephrological	25/327 (7.6%) (*p* = .478)	91/327 (27.8%) (*p* = .336)	2/43 (4.7%) (*p* = .478)	15/43 (34.9%) (*p* = .336)
Vascular	24/329 (7.3%) (*p* = .996)	98/329 (29.8%) (*p* = .170)	3/41 (7.3%) (*p* = .996)	8/41 (19.5%) (*p* = .170)
Gastrointestinal	22/298 (7.4%) (*p* = .957)	85/298 (28.5%) (*p* = .805)	5/72 (6.9%) (*p* = .957)	21/72 (29.2%) (*p* = .805)
Hepatic	24/346 (6.9%) (*p* = .276)	100/346 (28.9%) (*p* = .773)	3/23 (13.0%) (*p* = .276)	6/23 (26.1%) (*p* = .773)
Endocrine	18/269 (6.7%) (*p* = .449)	72/269(26.8%) (*p* = .238)	9/100 (9.0%) (*p* = .449)	33/100 (33.0%) (*p* = .238)
Autoimmune	27/353 (7.6%) (*p* = .236)	100/353 (28.3%) (*p* = .535)	0/17 (0.0%) (*p* = .236)	6/17 (35.3%) (*p* = .535)
Chronic infections	27/362 (7.5%) (*p* = .422)	104/362 (28.7%) (*p* = .817)	0/8 (0.0%) (*p* = .422)	2/8 (25.0%) (*p* = .817)

### Interdependence of influenza and *Streptococcus pneumoniae* vaccination

Results show a significant dependence between STIKO-compliant vaccination rates against *Streptococcus pneumoniae* and vaccination rates against influenza (*p* =  < 0.001; *V* = 0.213). The majority of the population was neither vaccinated against *Streptococcus pneumoniae* nor against influenza (68.6%), while only 4.6% were vaccinated against both (Table 4). More patients were vaccinated against influenza than against *Streptococcus pneumoniae* (24.1%). However, comparing the group of patients vaccinated against *Streptococcus pneumoniae* and analyzing within this group the proportion of patients vaccinated against influenza and those who were not, more patients were double vaccinated (4.6% vs. 2.7%). Of all patients vaccinated against *Streptococcus pneumoniae* according STIKO recommendation, only 31.8% were not vaccinated against influenza.

**Table 4:** Vaccination rates of *Streptococcus pneumoniae* and influenza in frequency (*n*/*N*) and percentage (%)

	No vaccination against *Streptococcus pneumoniae* (*n*/*N*) (%)	Vaccination against *Streptococcus pneumoniae n*/*N* (%)	Total *n*/*N* (%)
No vaccination against influenza *n*/*N* (%)	254/370 (68.6%) (*p* < .001)	10/370 (2.7%) (*p* < .001)	264/370 (71.4%)
Vaccination against influenza *n*/*N* (%)	89/370 (24.1%) (*p* < .001)	17/370 (4.6%) (*p* < .001)	106/370 (28.6%)
Total *n*/*N* (%)	343/370 (92.7%)	27/370 (7.3%)	370/370 (100.0%)

## Discussion

Risk of infections by respiratory pathogens is significantly increased for oncological patients. A 2014–2015 study of AGIHO (Working Group Infections in Hematology and Oncology in the German Society of Hematology and Oncology) participating centers showed that a high rate of lower respiratory tract infection (LRTI) and associated mortality is a relevant risk in this population group [23]. Depending on underlying cancer disease and antineoplastic treatment risk of IPD is up to 60-fold elevated [9]. The most common infections involved the respiratory tract and *Streptococcus pneumoniae* was one of the most prominent pathogens [24]. Vaccination could thus provide protection against *Streptococcus pneumoniae* as studies observed a sustainable antibody response even in immunocompromised patients [25].

For oncological patients hospitalized for influenza-related infections, a 9% chance of dying during their hospital stay has been observed [26], which is potentially preventable by annual vaccination. A meta-analysis showed a 70% decrease in the incidence of influenza-like illness in vaccinated compared to unvaccinated individuals [27]. A retrospective study of 1225 colorectal cancer patients undergoing chemotherapy found a lower incidence of pneumonia, lower 1-year mortality, and fewer treatment interruptions in vaccinated than unvaccinated patients [28]. These results underline the importance of vaccination in this population group. In light of these data, our results show that vaccination rates in Germany are in fact rather low. This is even less acceptable as German law requires physicians to offer vaccinations recommended by the STIKO [29].

Vaccination rates found in our study are unsatisfactorily low but in line with available data. A German retrospective study showed vaccination rates against *Streptococcus pneumoniae* in patients with “relevant diseases,” including cancer, ranged from 11.5 to 29.3%, with a mean of 19.0% [7]. Numbers are also similar to results of a French questionnaire survey reporting a vaccination rate against *Streptococcus pneumoniae* of 39%, but for this study, a substantial bias has to be taken into consideration, as participants were members of patients’ associations, significantly younger, 75% female, and exclusively with hematological malignancies [30]. Nonetheless, another French observational questionnaire study with oncological patients showed a similar coverage regarding vaccinations against *Streptococcus pneumoniae*, while documenting a positive attitude towards vaccination in 65%, demonstrating the scope of potential improvement [2].

Regarding influenza vaccination rates, it becomes apparent that other studies found higher vaccination rates among oncological patients. A study from the Netherlands showed a vaccination rate of 43.9% [31]. Data collected in 2016 and 2017 through the National Health Interview Survey of the USA indicated influenza vaccination rates among cancer patients of 64% [32]. Nevertheless, 64% were still rated as suboptimal and in need of significant improvement by the authors, which shows the strong necessity of increasing the rate of 28.1% identified in our study.

Our findings regarding the influence of gender on vaccination rates are concordant with the majority of studies. In general, as in our study, women show higher vaccination rates compared to men [33–36]. It is also striking that the group of patients over 60 is more frequently vaccinated against influenza. This result also corresponds to the findings of other studies [37]. This can be explained by the annually recurring general STIKO vaccination recommendation for the patient group over the age of 60 regardless of underlying illness [38]. Accordingly, the rate for vaccination with PPV23 alone was with 20.0% substantially higher than for sequential vaccination, but found only in the group of patients of age 60 years and older, following recommendations according age, not underlying disease. This could suggest that the general awareness for pneumococcal vaccination among treating physicians (both—as a vaccination due to age and as a vaccination due to oncological indication) is low. Indeed, in a French study evaluating patients with inflammatory bowel disease and GI cancers, the same phenomenon was observed; influenza vaccination is more prevalent compared to pneumococcal [20]. However, it should be noted that the study population over 60 years of age was significantly larger than the group under 60 years of age, which could possibly bias the result. The recent publication of new STIKO recommendations for pneumococcal vaccination, replacing all adult vaccination, including the sequential for immunocompromised, with a single shot 20-valent conjugate vaccine (PCV20), positively simplifies vaccination schemes and might result in better adherence [14].

The comparatively high vaccination rates against *Streptococcus pneumoniae* (14.6%) and influenza (39.0%) in patients with multiple myeloma compared with the other tumor diseases are noteworthy. A reason for this is probably the often chronic course of treatment of multiple myeloma, allowing vaccination in the course of time, and a standardized and clinically established vaccination regimen after stem cell transplantation. On the other side, none of the patients with acute or chronic leukemia has been vaccinated against influenza or *Streptococcus pneumoniae*, which might be related to the rapid disease progression and the treatment with intensive chemotherapy and/or CD20 antibodies.

Concerning and therefore worth mentioning are low vaccination rates, especially against *Streptococcus pneumoniae* (6.0%) but also against influenza (26.7%), in patients with thoracic carcinoma since these patients are extremely prone to IPD [8] and influenza [1]. Given the progress in the treatment of lung cancer with potentially long survival times even in the metastatic stage, it seems particularly important to increase vaccination rates in this subgroup.

In order to take into account, the influence of underlying secondary disease on *Streptococcus pneumoniae* and influenza vaccination rates, rates between patients with and without an underlying secondary disease were compared. The underlying hypothesis was that the presence of a secondary disease, such as a pulmonary chronic disease, could significantly augment the vaccination rate within this specific group, given their need for special protection. However, our findings revealed an opposing trend, especially among patients with pulmonary disease as a secondary diagnosis. Surprisingly, this subgroup exhibited a lower vaccination rate against both influenza and *Streptococcus pneumoniae*, despite the dual indication. One potential explanation for this phenomenon could be an amplified sense of caution among treating physicians when dealing with this patient cohort due to their heightened vulnerability. Consequently, there may be an erroneous presumption among treating physicians that vaccinations should be avoided for this group. Similar observations were also noted in patients with chronic infections as a secondary diagnosis.

Women (8.8% against *Streptococcus pneumoniae*; 36.8% against influenza) were vaccinated significantly more often than men (3.4% against *Streptococcus pneumoniae*, 16.9% against influenza). There is no clear explanation for the gender-specific differences in the rate of vaccination. The existing literature identifies various overarching barriers to vaccination, but none of these are specifically linked to gender. These barriers include factors such as perceptions of low disease risk, concerns about vaccine safety, and perceptions of perceived low benefit from vaccination [6]. Other studies (for example, on the dependence of vaccination rates against COVID-19 on gender) have shown the opposite results: men were significantly more likely to be vaccinated [39].

Our study shows a significant dependency between vaccination against influenza and against *Streptococcus pneumoniae*; more patients were vaccinated against both pathogens simultaneously. It can be assumed vaccination rates depend on individual information level of the treating general practitioner, also explaining the existing correlation between pneumococcal and influenza vaccination rates. Physicians sufficiently informed to vaccinate their patients against *Streptococcus pneumoniae* are more likely to vaccinate their patients against influenza as well. Anyhow, the opposite effect (patients vaccinated against influenza are also more likely to be vaccinated against *Streptococcus pneumoniae*) was not observed. Possibly because knowledge about annual influenza vaccination for those aged 60 + years is more widespread among treating physicians as well as among patients, in contrast to the age-related vaccination against *Streptococcus pneumoniae*. A study conducted in Stockholm, involving 10,000 participants, and specifically examining the correlation between influenza and pneumococcal vaccination rates is consistent with our findings. Similarly, most participants who received pneumococcal vaccination had also received influenza vaccination, while a significantly smaller proportion had received pneumococcal vaccination only [34].

## Limitations

As already mentioned above, data was collected during the COVID-19 pandemic, which affected the study in multiple ways. Firstly, access to health care was partly limited and patients might have avoided medical facilities, thus reducing potential for receiving vaccination. Temporary shortage of vaccines, especially against *Streptococcus pneumoniae*, may also have contributed to lower vaccination rates [40].

Study population was recruited in outpatient clinics in Berlin, Germany. Therefore, results are prone to both local and institutional influences. However, clinics are organized independently and based in different areas of Berlin. Adding to that, they represent a broad spectrum of patients and attending physicians. Nonetheless, socioeconomic data are lacking in our study, as they were not collected in the original trial. Therefore, a desirable deeper analysis of other potentially influencing factors was not possible.

Because of low absolute numbers of some subgroups and low numbers of vaccinated patients in general, statistics are more susceptible to bias.

## Conclusion

Despite the explicit recommendations and the critical need for vaccination protection among the high-risk population of oncological patients, a substantial proportion of these patients remain unvaccinated against both *Streptococcus pneumoniae* and influenza. New strategies are urgently needed to enhance vaccination rates for oncological patients. Addressing this issue requires focused interventions in three key areas: educating physicians, particularly general practitioners, about the significance and efficacy of vaccinations in this high-risk population; implementing standardized vaccination schedules for various oncological diagnoses based on the success with the EVO-Study [6]; and enhancing patient education to close the gap between recommended and actual vaccination status.

## Data Availability

Data are stored in the IT system of the Vivantes Hospital Neukoelln and available by request.
